# Supplementary material for: From sequence to enzyme mechanism using multi-label machine learning
Source: BMC Bioinformatics. 2014 May 19;15:150. doi: 10.1186/1471-2105-15-150 (PMC4229970; doi:10.1186/1471-2105-15-150)
Supplement: Additional file 2 — Java code of ml2db. Additional file ml2db_code.tar.gz contains the Java source code to run the multi-label machine learning experiments and save the results to database. The code’s Javadoc is included. [file 1471-2105-15-150-S2.zip › additional file 2/ml2db/ml2db/doc/main/ml2db/mulan/learn/traintest/class-use/TrainTestEvaluator.html]

Uses of Class main.ml2db.mulan.learn.traintest.TrainTestEvaluator


JavaScript is disabled on your browser.


- Overview
- Package
- Class
- Use
- Tree
- Deprecated
- Index
- Help

- Prev
- Next

- Frames
- No Frames

- All Classes

## Uses of Class main.ml2db.mulan.learn.traintest.TrainTestEvaluator

- Packages that use TrainTestEvaluator

  | Package | Description |
  | --- | --- |
  |  |  |
  | --- | --- |
  | main.ml2db.mulan.learn.traintest |  |
  | test.ml2db.mulan.learn.traintest |  |
- - ### Uses of TrainTestEvaluator in main.ml2db.mulan.learn.traintest

    Subclasses of TrainTestEvaluator in main.ml2db.mulan.learn.traintest

    | Modifier and Type | Class and Description |
    | --- | --- |
    |  |  |
    | --- | --- |
    | `class` | `TrainTestEvaluatorSerialised` Class |

    Methods in main.ml2db.mulan.learn.traintest that return TrainTestEvaluator

    | Modifier and Type | Method and Description |
    | --- | --- |
    |  |  |
    | --- | --- |
    | `TrainTestEvaluator` | TrainTestExperimenter.`getEvaluator()` |
    | `TrainTestEvaluator` | TrainTestExperimenter.`getNewEvaluator()` |
    | `TrainTestEvaluator` | TrainTestExperimenterSerialized.`getNewEvaluator()` |
  - ### Uses of TrainTestEvaluator in test.ml2db.mulan.learn.traintest

    Methods in test.ml2db.mulan.learn.traintest that return TrainTestEvaluator

    | Modifier and Type | Method and Description |
    | --- | --- |
    |  |  |
    | --- | --- |
    | `static TrainTestEvaluator` | TrainTestEvaluatorTest.`getMulanTrainTestEvaluatorBRk1NN()` |

    Methods in test.ml2db.mulan.learn.traintest with parameters of type TrainTestEvaluator

    | Modifier and Type | Method and Description |
    | --- | --- |
    |  |  |
    | --- | --- |
    | `static uk.ac.standrews.utils.main.database.TableRow` | TrainTestEvaluatorTest.`getEvaluationRow(TrainTestEvaluator evaluator, boolean predict, boolean saveTrueNegatives)` |

- Overview
- Package
- Class
- Use
- Tree
- Deprecated
- Index
- Help

- Prev
- Next

- Frames
- No Frames

- All Classes
